# Functional Characterization of MC1R-TUBB3 Intergenic Splice Variants of the Human Melanocortin 1 Receptor

**DOI:** 10.1371/journal.pone.0144757

**Published:** 2015-12-11

**Authors:** Cecilia Herraiz, Conchi Olivares, Maria Castejón-Griñán, Marta Abrisqueta, Celia Jiménez-Cervantes, José Carlos García-Borrón

**Affiliations:** Department of Biochemistry, Molecular Biology and Immunology, School of Medicine, University of Murcia, Campus de Espinardo, Murcia and IMIB-Arrixaca, El Palmar, Murcia, Spain; San Gallicano Dermatologic Institute, ITALY

## Abstract

The *melanocortin 1 receptor* gene (*MC1R*) expressed in melanocytes is a major determinant of skin pigmentation. It encodes a Gs protein-coupled receptor activated by α-melanocyte stimulating hormone (αMSH). Human *MC1R* has an inefficient poly(A) site allowing intergenic splicing with its downstream neighbour *Tubulin-β-III* (*TUBB3*). Intergenic splicing produces two MC1R isoforms, designated Iso1 and Iso2, bearing the complete seven transmembrane helices from MC1R fused to TUBB3-derived C-terminal extensions, in-frame for Iso1 and out-of-frame for Iso2. It has been reported that exposure to ultraviolet radiation (UVR) might promote an isoform switch from canonical MC1R (MC1R-001) to the MC1R-TUBB3 chimeras, which might lead to novel phenotypes required for tanning. We expressed the Flag epitope-tagged intergenic isoforms in heterologous HEK293T cells and human melanoma cells, for functional characterization. Iso1 was expressed with the expected size. Iso2 yielded a doublet of Mr significantly lower than predicted, and impaired intracellular stability. Although Iso1- and Iso2 bound radiolabelled agonist with the same affinity as MC1R-001, their plasma membrane expression was strongly reduced. Decreased surface expression mostly resulted from aberrant forward trafficking, rather than high rates of endocytosis. Functional coupling of both isoforms to cAMP was lower than wild-type, but ERK activation upon binding of αMSH was unimpaired, suggesting imbalanced signaling from the splice variants. Heterodimerization of differentially labelled MC1R-001 with the splicing isoforms analyzed by co-immunoprecipitation was efficient and caused decreased surface expression of binding sites. Thus, UVR-induced MC1R isoforms might contribute to fine-tune the tanning response by modulating MC1R-001 availability and functional parameters.

## Introduction

The melanocortin 1 receptor (MC1R), a major determinant of skin phototype, is a G protein-coupled receptor (GPCR) that regulates pigment production in melanocytes. When stimulated by α-melanocyte stimulating hormone or related peptides (the melanocortins, MCs) MC1R triggers cAMP synthesis leading to activation of the rate-limiting melanogenic enzyme tyrosinase. MC signaling also activates the MAP kinase module leading to ERK1 and ERK2, by a cAMP-independent mechanism involving transactivation of cKIT [1]. The cAMP and ERK pathways cooperate to regulate expression, activity and stability of Microphthalmia (MITF) transcription factor, a key positive modulator of melanocyte differentiation [2] that also regulates expression of the cell cycle regulatory proteins p21 and p27 [3, 4]. Increased tyrosinase activity in response to MC1R activation leads to the synthesis of dark eumelanin pigments as opposed to reddish pheomelanins, so as to increase the ratio of photoprotective eumelanins to pro-oxidant pheomelanins [5], thus providing an effective shield against mutagenic ultraviolet radiation (UVR). Moreover, MC1R orchestrates a complex series of events that coordinately improve antioxidant defenses and DNA repair mechanisms in UVR-exposed melanocytes (reviewed in [6]). In addition to direct effects on melanocytes, UVR activates transcription of the *POMC* gene in keratinocytes followed by release of MC peptides, thus achieving a paracrine stimulation of melanocytes [7].

**Fig 1:**
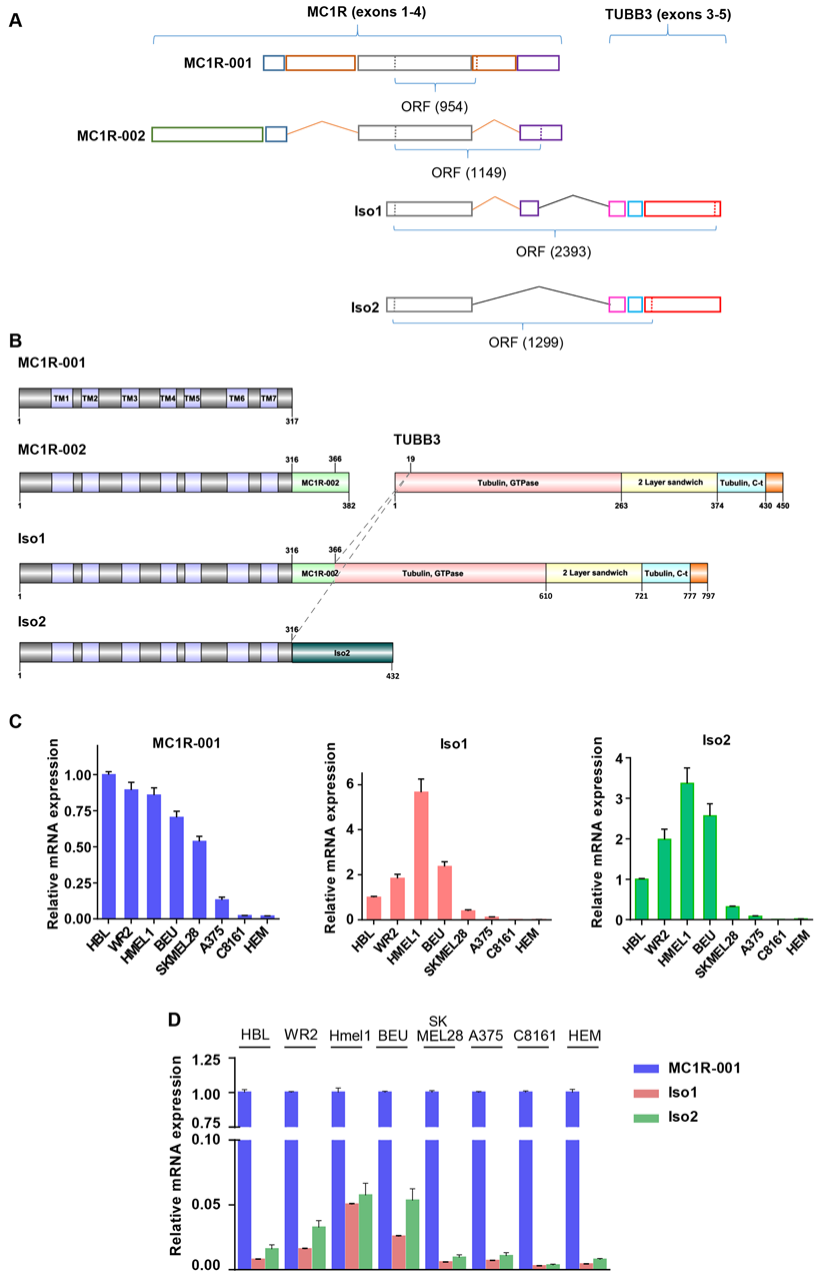
MC1R transcripts and intergenic splice isoforms of MC1R and TUBB3. (A) Schematic panel showing the exon organization of MC1R splice variants (MC1R-001 and MC1R-002) and MC1R-TUBB3 chimeric transcripts, Iso1 and Iso2. Exons of all MC1R derived transcripts are represented in colored boxes and the number of nucleotides in the ORF is shown below. (B) Diagram representing the structural domains of MC1R-001, MC1R-002, β-tubulin III (TUBB3) and chimeric proteins Iso1 and Iso2. Structural and functional domains are depicted in colored boxes and the number of key residues in the proteins is shown. TM indicates transmembrane regions of MC1R. Dashed lines indicate residues of MC1R and TUBB3 linked in fused proteins Iso1 and Iso2. (C) MC1R-001, Iso1 and Iso2 expression in human melanoma cell lines. Data are shown as relative expression of each isoform (as indicated in each bar graph) as compared with the levels of the isoform in HBL cells. (D) Expression of Iso1 and Iso2 mRNA as a function of the levels of the canonical MC1R-001 transcript in a panel of human melanoma cell lines. Data are represented as mRNA expression of the two intergenic splicing forms relative to MC1R-001 in each cell line.

The genes encoding for most GPCRs were thought to be most frequently intronless [8], but current evidence shows that over 50% of GPCR genes contain more than one intron [9]. The *MC1R* (MIM# 155555, ID ENSG00000258839) located at 16q24.3 is in fact quite complex as it contains 4 exons and yields several transcripts as a result of intra- and intergenic splicing, with usage of alternative splice donor-acceptor sites, retention of intronic sequences and skipping of exons and of translation termination and polyadenylation signals. The canonical 2.3 kb MC1R transcript containing a 951 nucleotides (nt) coding region [10] (Ensemble ID ENST00000555147, named MC1R-001) encodes for a 317 amino acid integral transmembrane protein with the typical structural characteristics of Class A GPCRs [9, 11], and contains exons 2, 3 and 4 with retention of unspliced intervening sequences located between exons 2–3 and 3–4 (Fig 1A and 1B). On the other hand, Tan and coworkers [12] reported an alternative spliced MC1R form designated MC1R-002 (ID ENST00000555427), which contains exons 1–4 resulting in a 1149 nt-long ORF encoding for a 382 amino acids protein. This splice isoform is identical to MC1R-001 up to Ser316, followed by an additional 65 amino acids C-terminal extension. Finally, the MC1R-003 transcript (ENST00000539976) lacks a functional open reading frame and is most likely a non-coding defective transcript.

In addition to these intragenic splice isoforms, a number of potentially functional intergenic splice variants involving the *MC1R* gene have been described [13]. These intergenic splice variants would arise as a result of the high gene density in the 16q24 region [10], where less than 8 kb separate the coding 3’ end of the next upstream gene and the *MC1R* initiation codon, and the intervening DNA fragment located between *MC1R* and the downstream *TUBB3* is only 2.5 kb-long. This dense packing, and the presence of an unusual and inefficient polyadenylation signal in human *MC1R* have been reported to promote intergenic splicing to the *TUBB3* gene [13, 14]. Two intergenic splice products have been described to date [13]. One of them contains *MC1R* exons 3 and 4 fused to *TUBB3* exons 3, 4 and 5 (Fig 1A). This transcript (*MC1R-TUBB3* gene, ID ENSG00000198211) encodes for a 797 amino acids in-frame fusion chimera named Iso1, corresponding to the first 366 residues of MC1R-002 and most of the TUBB3 sequence (Fig 1B). The other intergenic splice variant is an out-of-frame fusion of *MC1R* exon 3 and exon 3 of *TUBB3* (Fig 1A). The size of the predicted Iso2 protein product is 432 amino acids, with the first 316 amino acids matching the MC1R sequence. The remaining 116 C-terminal residues in this chimera share no homology with known proteins (Fig 1B) [13].

Since both Iso1 and Iso2 proteins virtually conserve all the structural elements in MC1R known to be important for agonist binding and coupling to downstream signaling pathways [6], they might retain a significant signaling potential. Interestingly, treatment of cultured melanocytes with αMSH or activation of p38-MAPK, both key molecules associated with UVR responses, shifts expression from MC1R-001 in favor of chimeric MC1R-TUBB3 isoforms [13]. Accordingly, the intergenic chimera might contribute to fine-tune the complex array of melanocytic adaptive responses to UVR insults orchestrated by the MC1R. However, their functional properties remain largely unknown. Here we report a study of the trafficking and signaling properties of the MC1R chimeric proteins that may shed light on their possible physiological role.

## Materials and Methods

### Materials

Igepal CA-630, BSA, EDTA, PMSF, iodoacetamide, bicinchoninic acid, anti-FLAG M2–Peroxidase conjugate antibody, anti-HA-peroxidase conjugate and anti-β-Tubulin III antibody were from Sigma (St. Louis, MO). The anti-pERK1/2 and anti-ERK2 rabbit polyclonal IgGs were from Santa Cruz Biotechnology (Santa Cruz, CA). The synthetic αMSH analogue [Nle4, D-Phe7]-αMSH (NDP-MSH) and the protein synthesis inhibitor cycloheximide were from Calbiochem (Darmstadt, Germany). The radioligand [^125^I]-NDP-MSH, specific activity 2000 Ci/mmol was from Amersham (Little Chalfont, Buckinghamshire, UK). The cAMP immunoassay kit was from Arbor Assays (Eisenhower Place, Michigan, USA). Lipofectamine 2000 was from Invitrogen (Carlsbad, CA). Reagents for SDS-PAGE and Western blot were from Bio-Rad (Richmond, CA, USA). Other reagents were from Merck (Darmstadt, Germany) or Prolabo (Barcelona, Spain).

### Cell culture, transfection and functional expression

HEK293T cells, PC12 cells and all human melanoma cells were grown in Dulbecco’s modified Eagle’s medium enriched with 10% fetal bovine serum, 100 U/ml penicillin and 100 μg/ml streptomycin sulfate.

All expression constructs were prepared in pcDNA3 (Invitrogen). The following expression constructs have been described: the Flag-tagged wild-type (WT) MC1R-001 [15], R151C and D294H [16], V60L and V92M [1]. N-terminal 3xHA-labeled MC1R-001 construct was from the Missouri University of Science and Technology cDNA Resource Center (Rolla, MO).

MC1R/TUBB3 locus constructs inserted into the pcDNA3.1-His expression vector were kindly provided by Prof A. Furger (University of Oxford, UK) and have been previously described [13]. To generate the Flag-tagged constructs, MC1R-TUBB3 chimeric transcripts were subcloned into pcDNA3 (Invitrogen) using EcoRI and XbaI as restriction enzymes. Site-directed mutagenesis using the QuickChange kit (Stratagene, La Jolla, CA) was performed to ablate BamHI restriction site within TUBB3 gene in pIso1 and pIso2 (primer pIso1: 5’-CTACTTCGTGGAGTGGATTCCCAACAACGTGAA-3’ and primer pIso2: 5’-CTACTTCGTGGAGTGGCTCCCCAACAACGTG-3’). N-terminal Flag epitope Iso1 and Iso2 constructs were obtained using a Flag-WT MC1R-001 as a template [17] and cleaving with BamHI and XbaI. Constructs were verified by automated sequencing in both strands.

HEK293T cells grown to approximately 80% confluence were transfected with 0.6 or 0.3 μg of plasmid DNA/well for 6 or 12-well plates, respectively, using Opti-MEM to dilute DNA and Lipofectamine (Invitrogen). HBL cells were transfected with 1 or 0.5 μg of plasmid DNA/well for 6 or 12-well plates, respectively.

### Binding and internalization assays

Radioligand binding assays have been described [17–19]. Cells grown in 12-well plates were transfected as required. Twenty-four hours after transfection, cells were serum deprived for 3 h and incubated with ^125^I-labelled NDP-MSH (5x10^-11^ M) and increasing concentrations of non-labelled competing NDP-MSH, from 10^−12^ to 10^−7^ M. To estimate internalization of agonist–receptor complexes, an acid wash procedure was used [20]. Cells were incubated (90 min, 37°C) with [^125^I]-NDP-MSH isotopically diluted to a final concentration of 10^−9^ M and 10^5^ cpm, washed with cold serum-free DMEM followed by two 2–3 min ice-cold acid washes with 0.5 ml of 50 mM glycine and 150 mM NaCl, pH 3.0. The acid washes were pooled and counted to determine the amount of non-internalized ligand bound on the cell surface. Cells were trypsin-harvested and counted for internalized receptor. Internalization indexes were defined as the percentage of internal relative to total ligand bound.

### Functional assays

For cAMP assays, HEK293T cells were grown in 12-well plates, transfected with MC1R-001 (WT, V60L, V92M, R151C or D294H), Iso1 or Iso2, serum-deprived for 24 h. They were then incubated with 10^-7^M NDP-MSH for 30 min. The medium was aspirated and the cells quickly washed with 800 μl ice-cold phosphate buffered saline (PBS). Stimulated cells were lysed with preheated 200 μl 0.1 N HCl (70°C) per well and scraped. The mix was freeze dried, washed with 100 μl H_2_O and freeze dried again twice. cAMP levels were measured by a commercial ELISA immunoassay, as per instructions. Briefly, samples and standards, a known concentration of cAMP-peroxidase conjugate, and a sheep antibody to cAMP were added into the wells of a microtiter plate coated with an antibody to capture sheep IgG. After washes the substrate was added. The substrate reacts with the bound cAMP-peroxidase conjugate and after a short incubation the reaction was stopped and the intensity of the generated color was detected at 450 nm in a BioTek™ ELx800™ microtiter plate reader (BioTek, Bedfordshire, UK). Parallel dishes were used for protein determination performed with the bicinchoninic acid method.

### Immunoprecipitation and Western blotting

Cells grown on 6-well plates were washed with PBS and solubilized at 4°C in 200 μl solubilization buffer (50 mM Tris-HCl pH 7.5, 1% Igepal, 1 mM EDTA, 0.1 mM PMSF, 10 mM iodoacetamide). Samples were centrifuged (21,000 g, 30 min). Suitable volumes of supernatant were mixed (2:1 ratio) with sample buffer (180 mM Tris-HCl, pH 6.8, 15% glycerol, 9% SDS, 0.075% Bromophenol Blue, 7.5% β-mercaptoethanol), electrophoresed and blotted as described [21]. Blots were probed with the required antibodies and stained with a chemiluminescent substrate (Amersham, Little Chalfont, UK). Comparable loading was ascertained by stripping and reprobing with an anti-ERK2 antibody. Stripping was performed by washing the membranes with PBS, followed by treatment with 0.5 N NaOH, 10 min at room temperature, and a final 10-min wash with H_2_O.

For immunoprecipitation, cells were washed, solubilized and centrifuged as above. Supernatants were incubated with 20 μl of EZview Red ANTI-FLAG M2 Affinity Gel beads (Sigma) for 1 h at 4°C. The mixture was washed, centrifuged, eluted with pre-heated (95°C) sample buffer, and electrophoresed and blotted as above.

### Confocal microscopy

Cells grown on coverslips were transfected, fixed with 4% paraformaldehyde in PBS and permeabilized with 0.05% Triton. Cells were labelled with anti-HA monoclonal (1:5000), followed by an Alexa 488 secondary antibody for detection of MC1R. For co-localization of HA-MC1R-001 and Flag-isoforms, cells were incubated simultaneously with anti-HA monoclonal and anti-Flag rabbit polyclonal, followed by Alexa 488-conjugated anti-mouse and Alexa 568-conjugated anti-rabbit secondary antibodies. Samples were mounted with a medium from Dako (Glostrup, Denmark) and examined with a Leica laser scanning confocal microscope AOBS (Leica Microsystems GmbH, Wetzlar, Germany). Images were taken in sequential scan mode between frames, with a HCX PL APO CS 63x objective. Co-localization analysis was performed in single cells using the line scan analysis of ImageJ.

### Flow cytometric analysis

Approximately 10^5^ cells were incubated in a final volume of 100 μl with anti-Flag M2 (1:25) for 30 min at 4°C. Cells were washed twice (2% fetal bovine serum, 0.01% NaN_3_ in PBS), and further incubated with a phycoerythrin-labelled anti-mouse IgG, at a final dilution of 1:50, for 30 min at 4°C. Cells were washed, resuspended in 500 μl PBS, and analyzed in a Becton Dickinson FACScan system.

### Real Time PCR

Several human melanoma cells were serum-deprived at least for 3 h before RNA extraction. Total RNA was extracted with the commercial kit RNeasy® Mini Kit (QIAGEN, Hilden, Germany) and measured with a NanoDrop 2000c spectophotometer (Thermo Scientific™ Waltham, Massachusetts, USA). One μg of RNA was reverse transcribed into cDNA using The SuperScript® III First-Strand Synthesis System for RT-PCR kit (Invitrogen Life technologies™, Carlsbad, California, USA) according to the manufacturer’s instructions.

qRT-PCR was performed using the Power SYBR Green PCR Master Mix (Applied Biosystem, Foster City, California, USA) on ABI 7500 Fast Real Time PCR System with the following cycling conditions: 95°C for 30 s and 40 cycles of 95°C for 5 s and 60°C for 60 s. The β-actin gene was used as the endogenous normalizer. Relative mRNA expression levels were calculated based on the Ct values using the formula 2^-ΔΔCt^.

Primers for MC1R-001 Iso1, Iso2 and β-actin, used after determination of their efficiencies, were provided by Biolegio BV (Biolegio BV, Nimega, Netherlands). Their sequences are:

MC1R-001 forward 5’-GCCCTCATCATCTGCAATGC-3’

MC1R-001 reverse 5’-CCCTCTGCCCAGCACACTTA-3’

Iso1 forward 5’-GCTCCTGCAAAAGGAGTTCTG-3’

Iso1 reverse 5’-GCTCGAGGCACGTACTTGTG-3’

Iso2 forward 5’-TGCTGACATGCTCCTGTTCTG-3’

Iso2 reverse 5’-GCTCGAGGCACGTACTTGTG-3’

β-actin forward 5'-GACAGGATGCAGAAGGAGATCA-3’

β-actin reverse 5'-GCTCAGGAGGAGCAATGATCTT-3’

### Statistical analysis

Unpaired two-tailed Student’s t-test and one-way ANOVA with Tukey post-test (for multiple comparisons) were performed using GraphPad Prism (GraphPad Software, San Diego California USA, www.graphpad.com). Data were presented as mean ± standard error mean (SEM). All p values were calculated using two-sided tests. P values of less than 0.05 were considered statistically significant. * indicates p<0.05, ** p<0.01, *** p<0.001 and **** p<0.0001.

## Results

### Expression of MC1R-TUBBIII splice variants

The Iso1 and Iso2 intergenic splicing chimerae were originally reported in HBL and M14 human melanoma cells [13]. Since gene expression patterns in human melanoma cell lines are very variable [22], it was of interest to compare the occurrence and abundance of the chimeric transcripts in a wider panel of melanoma cell lines of known genetic characteristics. This was approached by qRT-PCR (Fig 1C and 1D). We found detectable expression of the canonical MC1R-001 transcript in all cell lines, with large differences (~ 40-fold) between the cell lines with the highest and lowest levels (HBL and C8161 cells, respectively). The patterns of expression of Iso1 and Iso2 in the various cell lines were similar. However, when compared with MC1R-001, there where clear differences. For instance, the cell line expressing the highest level of MC1R-001 (HBL cells) was not the same as the one expressing more Iso1 and Iso2 (HMEL1 cells), and SKMEL28 cells with good expression of MC1R-001 had low levels of the intergenic spliced variants. Accordingly, the ratio of expression of chimeric mRNA normalized for expression of MC1R-001 was variable, with an approximately 20-fold difference between the cells expressing the highest and lowest ratios (HMEL1 and C8161, Fig 1D). Furthermore, similar analysis in human epidermal melanocytes (HEM) (cDNA kindly provided by Prof. J. Neptuno Rodríguez López, University of Murcia, Spain) revealed that basal expression of chimeric spliced variants in non-stimulated normal melanocytes was very low, comparable to the melanoma cell line C8161 expressing the lowest levels of Iso1 and Iso2 (Fig 1C). In addition, when intergenic spliced variants expression levels were normalized to MC1R-001 expression, we found that HEM expressed mainly the canonical MC1R-001 (Fig 1D).

**Fig 2:**
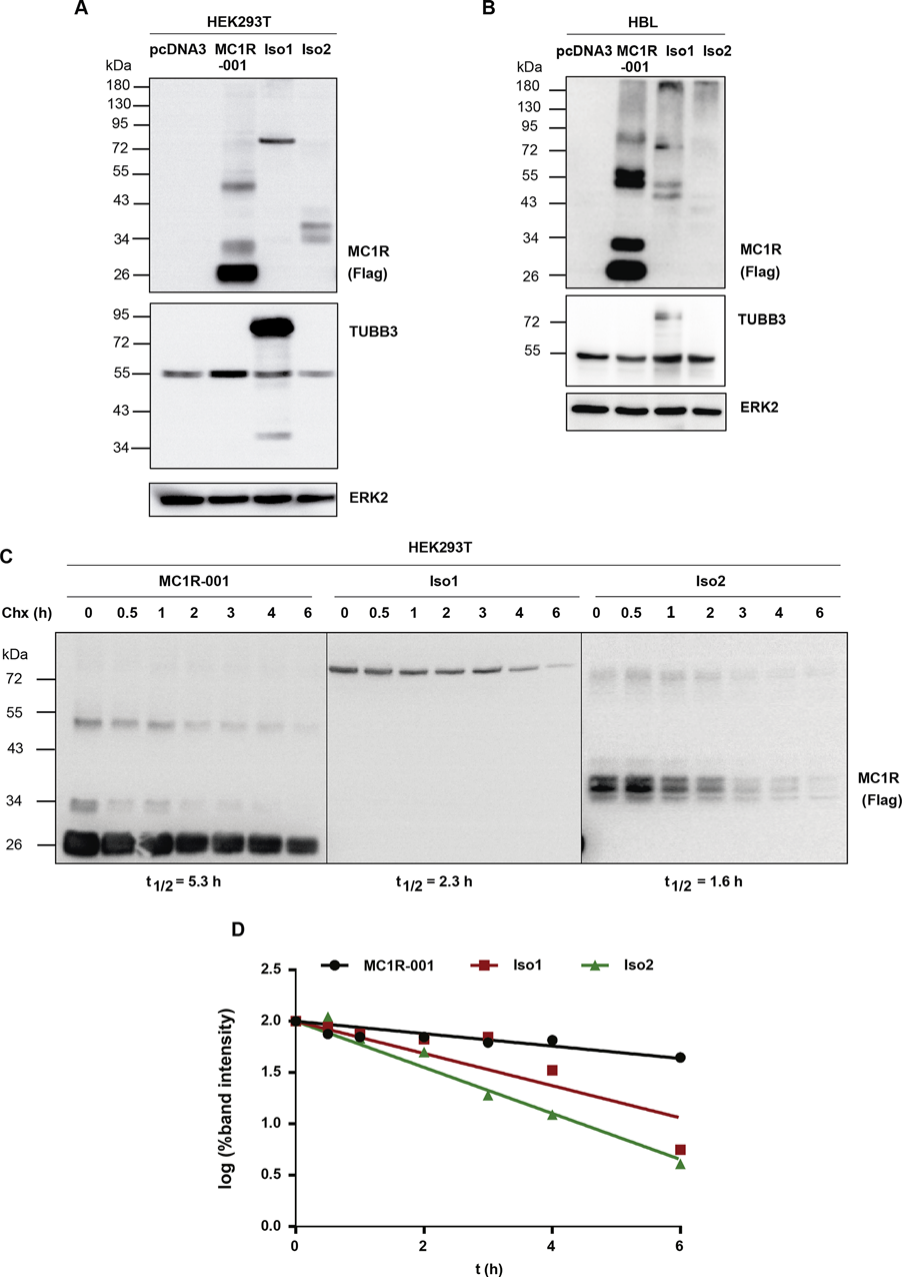
Electrophoretic analysis and intracellular stability of MC1R-TUBB3 isoforms. (A) Expression of canonical and chimeric MC1R proteins in heterologous HEK293T cells. HEK293T cells were transiently transfected to express Flag-labelled WT MC1R-001, Iso1 and Iso2. Cells were detergent-solubilized, electrophoresed and blotted. For MC1R detection, cell lysates were probed with an anti-Flag monoclonal antibody (upper blot). Membranes were also probed for TUBB3 (middle blot) and ERK2 (lower blot), as loading control (n = 5, representative blots are shown). (B) Electrophoretic pattern of MC1R-TUBB3 transcripts expressed in HBL human melanoma cells. Representative immunoblots for MC1R, TUBB3 and ERK2 are shown as in panel A (n = 5, representative blots are shown). (C) Intracellular stability of MC1R-TUBB3 chimeric fusion proteins in HEK293T cells. Flag-labelled MC1R-001, Iso1 and Iso2 were expressed in HEK293T cells. Cells were incubated with the protein synthesis inhibitor cycloheximide (Chx, 0.1 mM) for the times indicated, lysed and the levels of residual proteins in cell extracts were detected by Western blot. Representative immunoblots probed for MC1R-001, Iso1 or Iso2 with anti-Flag are shown. (D) Semi-log graph for calculation of half-lives. The intensity of receptor bands in the blots as in panel C was quantitated with ImageJ and the semi-log of residual signals was plotted against time. Half-life (t½) values correspond to the slope of the resulting lines.

MC1R-TUBB3 chimeric intergenic splice isoforms Iso1 and Iso2 contain the N-terminus and 7 transmembrane (TM) domains from MC1R (Fig 1A and 1B). Thus, they are potentially able to perform MC1R functions. In order to compare the signaling properties of the chimeric proteins and MC1R-001, we cloned Iso1 and Iso2 (with or without an N-terminal fused Flag epitope) and expressed them in heterologous HEK293T cells, along with the canonical receptor. When expressed in HEK293T cells (Fig 2A), as previously reported [1, 16], MC1R migrated as a doublet with a majority band of apparent Mr ~ 29 kDa and a minority band of ~ 34 kDa, corresponding to *de novo* WT MC1R-001 and an EndoH-sensitive glycoform, respectively. Moreover, Iso1 migrated with the expected apparent molecular weight (Mr ~ 88 kDa) whereas Iso2 showed a Mr around 38 kDa, lower than predicted. As expected, the Iso1 in-frame fusion of MC1R and TUBB3 cross-reacted with anti-TUBB3 antibodies (Fig 2A) whereas the out-of-frame chimera Iso2 did not (blot below). Comparison of band intensities for the Flag-tagged proteins showed lower steady-state levels of Iso1 and Iso2 compared with WT MC1R-001, suggesting a lower intracellular stability for the chimeric forms. A higher rate of intracellular proteolysis would also be consistent with the Mr of Iso2, lower than expected on the basis of its predicted amino acid sequence, as well as with the finding of more than one discrete immunoreactive band. We further analyzed the electrophoretic pattern of Iso1 and Iso2 intergenic splicing chimeras expressed in the human melanoma cell line HBL (Fig 2B). We found a similar pattern of migration for Iso1, with an apparent Mr of 88 kDa and cross-reactivity with TUBB3 antibody. However, Iso2 was detected as a faint band of very high Mr, suggesting protein aggregation and/or ubiquitylation followed by degradation.

Therefore, we tested the possibility of a higher intracellular proteolytic processing by following the decay of MC1R-001, Iso1 and Iso2 in HEK293T cells treated with the protein synthesis inhibitor cycloheximide (Fig 2C and 2D). The decay rate of the proteins was faster for Iso2, intermediate for Iso1 and slower for MC1R-001, corresponding with half-life values of 1.6, 2.3 and 5.3 h, respectively (Fig 2C and 2D) consistent with the steady state protein levels.

### Agonist binding and cell surface expression of MC1R-TUBBIII splice variants

**Fig 3:**
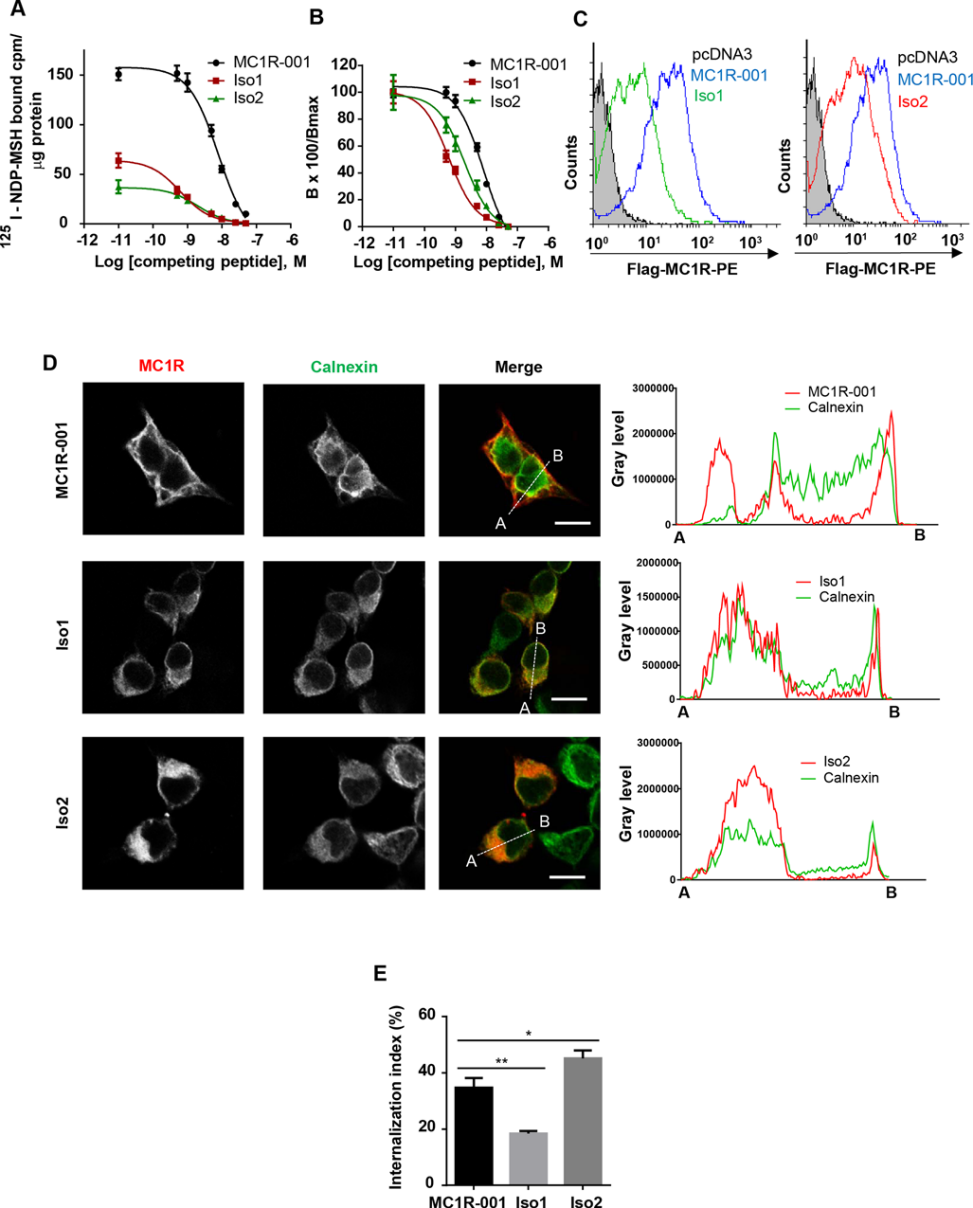
Radioligand binding and intracellular trafficking properties of MC1R-TUBB3 isoforms. (A-B) Competition binding assay of HEK293T cells transfected with MC1R-001, Iso1 and Iso2. Cells were incubated with ^125^I-labelled NDP-MSH (5x10^-11^ M) and increasing concentrations of non-labelled competing NDP-MSH, from 10^−12^ to 10^−7^ M, extensively washed and counted for radioactivity. Non-specific binding was determined with non-transfected cells or with transfected cells incubated with the radioactive tracer in the presence of excess (10^−6^ M) non-labelled peptide, with the same results. Values are represented as specifically bound [^125^I]-NDP-MSH (A) and as percentage of residual binding (B) at the different ligand concentrations (n = 3, data are given as mean ±SEM). (C) Flow cytometric analysis of HEK293T cells expressing MC1R-001 and MC1R-TUBB3 chimeric isoforms. Non-permeabilized cells expressing the indicated proteins were incubated with an anti-Flag antibody labelled with phycoerythrin. Since the Flag epitope was fused in-frame to the extracellular N-terminus of the MC1R sequence, only cells expressing the constructs of the plasma membrane should be detected. Histograms represent cell number (counts) as a function of Flag surface staining, on a logarithmic scale. The gray filled curve refers to cells transfected with an empty pcDNA3 (n = 3, representative histograms are shown). (D) Left panel: Representative confocal images of MC1R-001 or the chimeric isoforms (red) and calnexin (green) immunostaining in HEK293T cells transiently transfected with Flag-labelled MC1R-001 and MC1R-TUBB3 constructs. Scale bar, 10 μm. Representative line scan (right panel) from multiple experimental repeats across the cell (location indicated in merged image) shows co-localization of MC1R-TUBB3 transcripts and calnexin. Line scan, 19 μm for MC1R-001, 17 μm for Iso1 and 18 μm for Iso2. (E) Radioligand internalization assay performed on HEK293T cells expressing MC1R-001, Iso1 or Iso2 incubated with ^125^I-labelled NDP-MSH. The radioactive tracer was isotopically diluted to achieve a final concentration of 5x10^-11^ M and 5x10^4^ counts/well. Externally bound agonist was separated by an acid wash procedure. Both the externally bound ligand present in the acid washes and the internalized ligand associated with the cell pellets were counted. The internalization index represents the percentage of ligand internalized referred to total radioligand bound (n = 3, error bars are ±SEM, two-sided one-way ANOVA was used to generate p values, *p<0.05, **p<0.01).

We compared agonist binding parameters for MC1R-001, Iso1 and Iso2 expressed in HEK293T cells, by equilibrium binding assays using [I^125^]-NDP-MSH as the radioactive tracer and unlabelled NDP-MSH as competing peptide. The data were plotted as specific binding normalized for protein to compare maximal levels of bound agonist in cells expressing each variant (Fig 3A) and as % of maximal binding to each isoform for an easier comparison of the relative affinity of the variants (Fig 3B). Both Iso1 and Iso2 bound radiolabelled agonist specifically, but maximal binding was low, with residual Bmax values of about 10% (Table 1). However, affinity remained high with IC50 and Kd values in the low nM range (Fig 3A and 3B and Table 1). In fact, the affinity for NDP-MSH was even slightly higher for the chimeric forms, as shown by left-shifted displacement curves (Fig 3B).

**Table 1:** Binding parameters of WT MC1R-001 and chimeric transcripts Iso1 and Iso2.

MC1R transcript	Bmax (fmoles/mg protein)	Kd (nM)
MC1R-001	3.47 ± 0.18	1.99 ± 0.49
Iso1	0.27 ± 0.03	0.59 ± 0.27
Iso2	0.37 ± 0.04	1.17 ± 0.52

Equilibrium binding parameters of [^125^I]-NDP-MSH to MC1R-001 or the MC1R-TUBB3 isoforms Iso1 and Iso2 expressed in HEK293T cells (n = 3, data are given as mean ±SEM).

These binding parameters suggested aberrant intracellular trafficking of MC1R-TUBB3 chimeric proteins with decreased cell surface expression of the fusion proteins. This was further tested by flow cytometry (Fig 3C). Non-permeabilized cells were stained with an anti-Flag antibody directed against the Flag epitope fused to the extracellular N-terminus of the protein. In these non-permeabilizing conditions, only receptor molecules inserted on the plasma membrane with the correct orientation should be detected. The intensity of staining was much lower for the chimeric proteins compared with WT receptor. Lower plasma membrane levels of Iso1 and Iso2 could in turn result from an inefficient forward movement or from an increased rate of sequestration away from the cell surface. Forward trafficking was assessed by confocal microscopy analysis of co-localization with calnexin, an endoplasmic reticulum (ER)-resident chaperone. Extensive co-localization with calnexin was found for Iso1 and Iso2, whereas expression of the isoforms on the cell surface was almost undetectable (Fig 3D). Conversely, co-localization of calnexin and MC1R-001 was much lower and presence of the receptor on the plasma membrane was easily detected. These results indicated massive intracellular retention and failure to escape the quality control mechanisms of the secretory pathway for Iso1 and Iso2 proteins. On the other hand, retrograde transport away from the cell surface was estimated by an acid-wash procedure that allows distinguishing external (acid-sensitive) binding sites and internalized (acid-resistant) radioligand-receptor complexes (Fig 3E). Internalization of Iso1 was also significantly impaired. Conversely, Iso2 was internalized at slightly higher rates than MC1R-001, which may contribute to its low cell surface expression.

### Functional coupling

**Fig 4:**
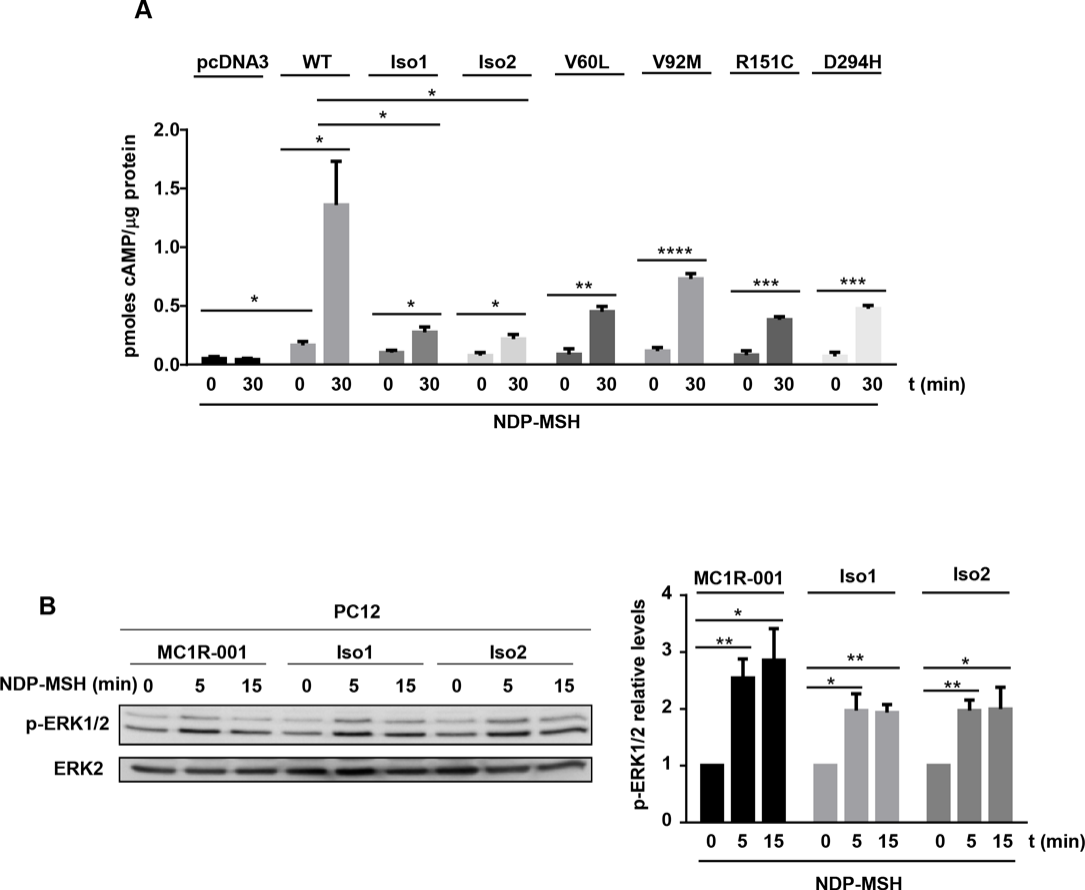
Effects of intergenic splicing on the functional coupling of MC1R to the cAMP and ERK1/2 pathways. (A) Agonist-induced cAMP production in HEK293T cells expressing MC1R-001, Iso1, Iso2, or the natural MC1R-001 variant allelesV60L, V92M, R151C and D294H. Cells were incubated with 10^−7^ M NDP-MSH for 30 min and cAMP levels were determined by an immunoassay (n = 6, error bars are ±SEM, two-sided Student´s t test was used to generate p values, *p< 0.05, *** p< 0.001). (B-C) Representative immunoblots (B) and quantification (C) of ERK1 and ERK2 phosphorylation in PC12 cells transfected to express MC1R-001, Iso1 or Iso2 and stimulated with NDP-MSH (10^−7^ M) for the times indicated (n = 5, error bars are ±SEM, two-sided Student´s t test was used to generate p values, *p< 0.05, ** p< 0.01).

Activation of MC1R potently stimulates cAMP synthesis, and triggers ERK signaling through a cAMP-independent pathway [1, 23]. We tested the chimeric proteins for activation of the cAMP and ERK pathways. For comparison, residual signaling from frequent hypomorphic MC1R variants associated with a red hair color phenotype with lower (“r” variants V60L and V92M) or higher penetrance (“R” forms R151C and D294H) was also estimated. HEK293T cells were transfected with the MC1R forms, challenged with a saturating concentration of NDP-MSH for 30 min, and cAMP contents were determined. Functional coupling of Iso1 and Iso2 to the cAMP pathway was strongly impaired relative to MC1R-001, as shown by lower cAMP production upon stimulation with NDP-MSH (Fig 4A). The residual signaling potential of the chimeric fusions was lower than V60L and V92M, and comparable with R151C and D294H. On the other hand, MC1R activates the mitogen-activated protein kinases ERK1 and ERK2 by a cKIT transactivation mechanism independent of cAMP, which is less sensitive to many natural mutations than activation of the cAMP cascade [24]. We analyzed ERK activation downstream of Iso1 and Iso2 in PC12 cells transfected to express the canonical or the chimeric receptors (Fig 4B). Signaling from the intergenic splice isoforms to ERK activation showed a decreasing trend compared with MC1R-001, but the differences in the maximal levels of active ERKs did not reach statistical significance (Fig 4B).

### Functional interactions of WT MC1R and intergenic splice variants

**Fig 5:**
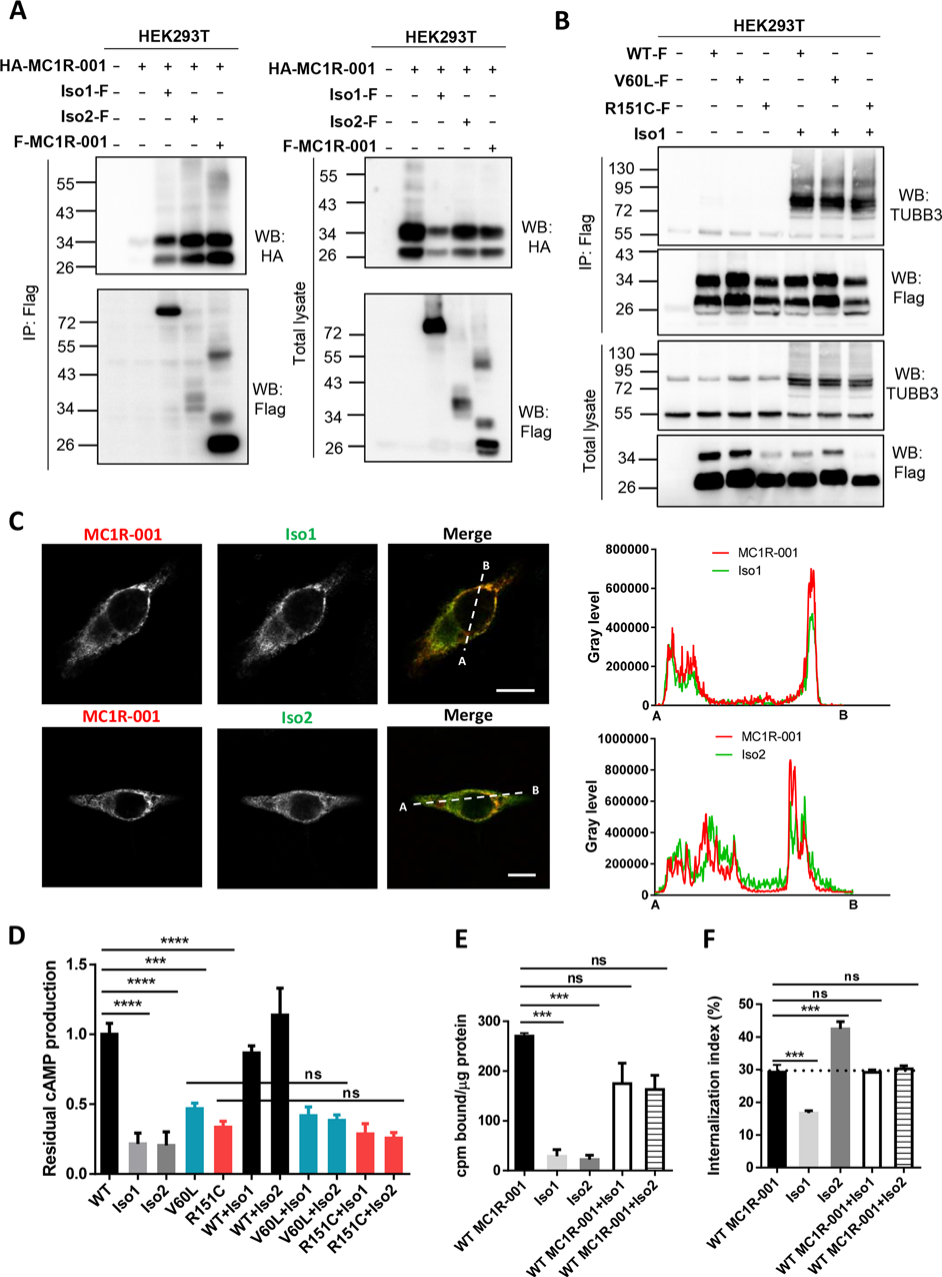
Heterodimerization of MC1R and MC1R-TUBB3 chimeric isoforms. (A) Co-immunoprecipitation of MC1R-001 and the MC1R-TUBB3 chimeric isoforms. HEK293T cells expressing the indicated constructs were lysed and immunoprecipitated for Flag-labelled MC1R-001, Iso1 or Iso2 using an anti-Flag monoclonal antibody. The pellets were electrophoresed and blotted for HA-labelled MC1R-001 (with a specific anti-HA monoclonal antibody) or for Flag-labelled MC1R-001, Iso1 or Iso2 (with anti-Flag monoclonal antibody) as a control for efficient immunoprecipitation. Total lysates were also electrophoresed and blotted as expression controls (n = 3, representative blots are shown). (B) Co-immunoprecipitation of V60L or R151C variant MC1R-001 and WT MC1R-TUBB3 intergenic splice isoform Iso1. The indicated constructs were expressed in HEK293T cells and immunoprecipitated for Flag-labelled MC1R-001, V60L or R151C using an anti-Flag monoclonal antibody. Immunoblots for Flag-tagged constructs and TUBB3 are shown for immunoprecipitated and total lysates. (C) Representative confocal images of MC1R-001 (green) and Iso1 or Iso2 (red) immunostaining in HBL cells transiently transfected with HA-labelled MC1R-001 and Flag-labelled MC1R-TUBB3 chimeric isoforms. Scale bar, 10 μm. Representative line scan (right panel) from multiple experimental repeats across the cell (location indicated in merged image) shows co-localization of canonical MC1R-001 and chimeric proteins. Line scan, 20 μm for MC1R-001+Iso1, and 31 μm for MC1R-001+Iso2. (D) Effect of heterodimerization on functional coupling to cAMP. Intracellular cAMP levels in HEK293T cells expressing WT, V60L or R151C MC1R-001 alone or in combination with Iso1 and Iso2 upon stimulation with 10^−7^ M NDP-MSH for 30 min. Results are presented as residual cAMP production relative to WT MC1R-001 (for which cAMP levels were 0.096±0.043 and 0.889±0.071 pmol/μg protein in resting and stimulated conditions respectively) (n = 3, error bars are ±SEM, two-sided one-way ANOVA was used to generate p values *p< 0.05, **p< 0.01, ***p< 0.001). (E) Specific binding of [^125^I]-NDP-MSH (5x10^-11^ M and 5x10^4^ cpm) to HEK293T cells expressing MC1R-001 or the MC1R-TUBB3 isoforms Iso1 and Iso2, alone or in combination (n = 3, error bars are ±SEM, two-sided one-way ANOVA was used to generate p values, ***p< 0.001). (F) Agonist internalization index in HEK293T cells co-transfected with MC1R-001 and Iso1 or Iso2 upon incubation with [^125^I]-labelled NDP-MSH (5x10^-11^ M and 5x10^4^ cpm) for 90 min (n = 3, error bars are ±SEM, two-sided one-way ANOVA was used to generate p values, ***p< 0.001).

We have previously shown that MC1R exists as dimeric species [21, 25], and that heterodimerization of WT and mutant forms gives raise to dominant negative effects [21, 26]. Since dimerization apparently proceeds through a domain swap mechanism involving the 7 TM fragments expressed in Iso1 and Iso2 [25], *in vivo* formation of MC1R/Iso heteromeric species is likely. We analyzed the occurrence of heterodimerization by co-immunoprecipitation of differentially epitope-labelled variants. First, MC1R-001 tagged by in frame fusion of the HA epitope to its N-terminus, and chimeric proteins (or MC1R-001 as positive control) labelled at the N-terminus with the Flag epitope were expressed alone or in combination in HEK293T cells. The intergenic chimeras were immunoprecipitated from detergent-solubilized extracts with anti-Flag agarose beads, and the pellets were analyzed for MC1R-001 by Western blot probed with anti-HA (Fig 5A). Co-immunoprecipitation of MC1R-001 and the MC1R-TUBB3 chimeric proteins was readily detected, indicating efficient heterodimerization. In addition, to mimic a heterocygotic MC1R genetic background highly frequent in northern European population, we tested the heterodimerization capability of two common hypomorphic variant MC1R alleles with the WT MC1R-derived chimeric protein Iso1. We selected the frequent V60L and R151C alleles as representative of the r and R types of RHC alleles, respectively. Flag-labelled versions of these constructs were overexpressed alone or with intergenic splice variant Iso1 in HEK293T cells. The amount of chimeric protein Iso1 immunoprecipitated with MC1R was comparable for WT and the variant alleles V60L and R151C (Fig 5B). Therefore, in a RHC variant allele background, heterodimerization between MC1R variant alleles and splice variants Iso1 and Iso2 may occur.

Moreover, we examined the intracellular localization of MC1R-001 and Iso1 or Iso2 by confocal microscopy in HEK293T cells co-expressing MC1R-001 and one of the intergenic splice forms (Fig 5C). We found a high degree of co-localization of MC1R-001 and Iso1 or Iso2 in internal compartments, suggesting that heterodimerization impairs forward trafficking compared with MC1R-001 homodimers. Nevertheless, we also detected co-localization of MC1R-001 and Iso1 or Iso2 at the cell periphery, consistent with higher expression of the chimeric proteins on the cell surface when co-expressed with MC1R-001 compared with cells expressing the isoforms alone.

We next estimated agonist-induced cAMP production in HEK293T cells co-expressing WT or variant alleles V60L or R151C and chimeric proteins Iso1 and Iso2 (Fig 5D). The cAMP response was similar in cells expressing WT or variant alleles MC1R-001 alone, or MC1R-001 and the chimeric forms. On the other hand, co-expression of canonical and the chimeras slightly decreased cell surface expression of binding sites, although the differences did not reach statistical significance (Fig 5E). Conversely, no effects on internalization rates were detected, with comparable results for cells expressing WT MC1R-001 alone, or in combination with Iso1 or Iso2 under conditions previously shown to result in efficient heterodimerization (Fig 5F).

## Discussion

Recent progress in the analysis of the architecture of genes encoding for GPCRs has shown that they often contain several introns and can undergo alternative splicing events. However, intergenic splicing giving raise to chimeric molecules is a rare event [27] where transcription proceeds through the region between two adjacent genes to yield a non-canonical chimeric RNA which is further spliced to a final fusion product composed of sequences from the two neighboring genes. To the best of our knowledge, this process has been described only for the P2Y receptor and the SSF1 genes [28] on one hand, and the *MC1R* and *TUBB3* genes on the other within the large GPCR superfamily [13, 14].

We confirmed the occurrence of Iso1 and Iso2 MC1R-TUBB chimeric mRNA species in a panel of 8 human melanoma cell lines and a human epidermal melanocytic cell line. We were able to detect the corresponding transcripts in all of them even in the absence of external stimuli, thus showing that expression of Iso1 and Iso2 is a general phenomenon in human melanocytic cells. We also observed that the ratio of Iso1 and Iso2 transcripts relative to canonical MC1R-001 transcript was variable in different cell lines. This suggests that Iso1/2 may not merely result from an unregulated error of the transcriptional machinery occurring at a constant rate within the context of the crowded genomic region where *MC1R* is located. Should this be the case, a linear relationship between MC1R-001 and Iso1/2 mRNA would be expected. In keeping with these data, Dalziel and co-workers reported that Iso1/2 expression is a regulated process, activated by αMSH and by the p38 stress activated kinase [13].

When expressed in HEK293T heterologous cells or in human melanocytic cells, the in-frame Iso1 intergenic fusion protein exhibited the expected Mr and cross-reacted with anti-TUBB3 antibodies. Therefore, chimeric mRNA was adequately processed and the resulting protein accumulated at sufficient levels within transfected cells, although its intracellular stability was lower than the one of MC1R-001. On the other hand, Iso2 results from an out-of-frame fusion with *TUBB3* exon 3, skipping exon 1a in the MC1R 3’-UTR. Hence, Iso2 did not cross-react with anti-TUBB3 antibodies. Moreover, its intracellular levels and half-life were low. The shorter half-life of the chimeric proteins was consistent with impaired forward trafficking demonstrated by reduced cell surface expression and high co-localization with the ER marker calnexin. Thus, the ER-resident protein quality control system most likely recognized the chimeric proteins as aberrant, causing their ubiquitylation, extrusion to the cytosol and proteolytic degradation [29].

Both chimeras exhibited very poor ability to activate the cAMP pathway. This might be at least partially due to decreased cell surface expression of the chimeric receptor compared to WT MC1R. Residual cAMP levels after stimulation with a saturating concentration of the superpotent NDP-MSH analog of αMSH were lower than those obtained in cells expressing known red-hair color-associated MC1R variants with low (V60L and V92M) or high (R151C and D294H) penetrance [24]. Conceivably, chimeric proteins arising from variant MC1R may be even more severely reduced in signaling capacity, but we did not test directly this likely hypothesis. Functional impairment was nevertheless less evident for activation of the ERK cascade. Accordingly, signaling from the MC1R-TUBB3 intergenic splicing isoforms seemed biased in favor of the ERK pathway. A similar behavior has been shown for many natural MC1R variant alleles associated with the red hair color phenotype, with increased skin cancer risk [24], higher mean survival of patients [30, 31] and the anatomic site presentation of melanomas [32]. Therefore, when expressed following exposure to UVR, the chimeric isoforms might prevent excessive stimulation of the cAMP pathway without a parallel decrease in melanocortin-dependent ERK activation. This effect might be enhanced by the ability of the isoforms to heterodimerize with MC1R-001, which seemed to impair forward trafficking of the canonical form and lead to its partial intracellular retention. This was suggested by high co-localization of the canonical protein and the chimeric isoforms in intracellular locations as well as a by an apparently decreased number of αMSH binding sites on the cell surface of cells overexpressing both proteins simultaneously. Although in this case the observed decrease did not reach statistical significance, it appears likely that under normal expression conditions it may become a significant factor.

The observation that expression of the intergenic splicing isoforms is a general feature in human melanoma cell lines (this manuscript) as well as a regulated process [13] strongly suggests that Iso1 and Iso2 might fulfil still uncharacterized specific actions within melanocytes. Given that both isoforms are hypomorphic in terms of functional coupling to the cAMP pathway, these actions might likely be related with dampening MC1R signaling under specific physiological conditions. UVR-mediated upregulation of MC1R molecules on the cell surface of melanocytes has been shown to occur at least partially by transcriptional activation of the *MC1R* gene. Direct UVR-mediated upregulation of *MC1R* expression has been shown in mouse and human melanocytic cells [33, 34] as well as in human epidermis *in vivo* [35]. In addition, *MC1R* gene expression is upregulated, apparently via MITF, in human and mouse melanocytes stimulated with αMSH or the cAMP inducer forskolin [36–38]. UVR-induced DNA damage in keratinocytes has been shown to stabilize the p53 tumor suppressor, which activates transcription of *POMC* gene encoding for the precursor of αMSH. αMSH is then released from keratinocytes to activate MC1R in melanocytes [39]. Accordingly, UVR would increase MC1R expression by at least two types of processes, namely a direct stimulation of MC1R gene expression in irradiated melanocytes on one hand, and, on the other, an indirect pathway whereby release of POMC-derived MC1R agonists by keratinocytes in UVR-exposed skin would activate MC1R signaling in melanocytes, followed by increased cAMP signaling and induction of *MC1R* transcription. This would further increase the responsiveness of melanocytes to the paracrine signals resulting in a positive feedback loop that may favor a potent tanning response, but that may also threaten melanocyte viability owing to the inherent cytotoxicity of the melanogenic pathway [40, 41]. It has been shown that Iso1/2 expression increases following stimulation of melanocytes with αMSH or activation of the p38 kinase, in an isoform switch that favors their expression relative to the canonical transcript [13]. Thus, the same signaling cascades implicated in induction of *MC1R* gene expression have also been shown to promote Iso1 and Iso2 transcripts. The diversion of a fraction of the new transcriptional events towards formation of inactive, and maybe even dominant-negative intergenic splice isoforms might endeavor the melanocytes with a mechanism to dampen and fine-tune this potentially dangerous positive feedback loop.
